# Among Ectasia Patients with Coexisting Coronary Artery Disease, TIMI Frame Count Correlates with Ectasia Size and Markis Type IV Is the Commonest

**DOI:** 10.1155/2015/282170

**Published:** 2015-02-03

**Authors:** Hasahya Tony, Kai Meng, Bangwei Wu, Qiutang Zeng

**Affiliations:** ^1^Institute of Cardiology, Union Hospital, Tongji Medical College, Huazhong University of Science and Technology, Avation Road 13, Wuhan 43003, China; ^2^Department of Cardiology, The Second Hospital of Shandong University, Jinan, Shandong 250033, China; ^3^Department of Cardiology, Huashan Hospital, Shanghai Medical College, Fudan University, Shanghai 200040, China

## Abstract

*Background*. Coronary artery ectasia (CAE) occurs in 0.3 to 5.3% of patients undergoing coronary angiography. TIMI frame count (TFC) is an index of coronary flow that correlates with flow velocity. In ectasia patients, there is delayed coronary flow with increased TFC. *Methods.* We evaluated angiograms of 789 patients for presence of CAE, coronary artery disease (CAD), and Markis type of CAE. We measured ectasia size and length and their correlation with TFC in ectatic right coronary arteries (RCA) of patients with CAE and CAD. *Results.* 30 patients had CAE (3.8%). Of these 16.7% had isolated CAE, while 83.87% had CAE and CAD. Among CAE and CAD patients, the RCA was most involved (70.4%), and Markis type IV CAE was the commonest (64%). In isolated CAE, the RCA, LAD, and LCx were equally involved (33.3%). Patients with CAE and CAD had significantly higher TFC compared to controls, *P* = 0.035. There was a positive correlation of moderate strength, between ectasia size and TFC, *r*(17) = 0.598, *P* = 0.007. Ectasia length was not significantly correlated with TFC, rho (17) = 0.334, *P* = 0.163. *Conclusion.* Among patients undergoing angiography, CAE has a prevalence of 3.8% and Markis type IV is the commonest. Larger ectasias are associated with slower coronary flow.

## 1. Introduction

Coronary artery ectasia is the abnormal dilatation of a segment of coronary artery to 1.5 times or more the size of adjacent normal segment of the artery [1–4]. The incidence of coronary artery ectasia has been reported to be 0.3 to 5.3% of patients undergoing coronary angiography [1–4].

20 to 30% of cases of coronary ectasia are considered congenital and the others are acquired. Of the acquired cases, 50% are attributed to atherosclerosis while 10% to 20% are associated with inflammatory and connective tissue diseases (like Ehlers-Danlos syndrome, Kawasaki disease, and scleroderma), syphilis, and bacterial infections [1–4].

The most common clinical presentation is angina. Other presentations include ST-elevation myocardial infarction, non-ST-elevation myocardial infarction, arrhythmias, spontaneous dissection of an ectatic coronary artery, and sudden death [4].

TIMI frame count is an index of coronary flow as a continuous quantitative variable. TIMI frame count measurements are correlated with flow velocity measured with a flow wire during baseline and hyperemia [5]. Studies have shown that there is delayed coronary flow with increased TIMI frame counts in coronary ectasia patients [5, 6].

Because CAE renders patients to higher risk of myocardial ischemia irrespective of the extent of stenosis, more studies characterizing this understudied disease entity are necessary to further improve management. We believe our study will add to the existing knowledge about this pathology.

## 2. Methodology

We carried out a retrospective study, and our objectives were to establish the prevalence of coronary artery ectasia and its sex distribution among patients undergoing coronary angiography, analyze the occurrence of different ectasia types according to Markis classification, assess coexisting obstructive coronary artery disease (CAD) among ectasia patients, and assess TIMI frame count and the effect of ectasia size and length on the TIMI frame count in these patients.

### 2.1. Study Population

The study group consisted of 30 patients found to have coronary ectasia among the 789 consecutive patients we reviewed, who underwent coronary angiography for various indications, by the Judkins technique, from January 1, 2010, to June 30, 2010, in the Department of Interventional Cardiology, Union Hospital of Tongji Medical College of Huazhong University of Science and Technology, Wuhan, China. Of these, 25 patients had coronary ectasia and coexisting obstructive coronary artery disease and 5 had isolated coronary ectasia. The control group consisted of 12 age and sex matched patients with angiographically proven normal coronary arteries. The patient clinical characteristics including age, sex, smoking status, diabetes mellitus, and hypertension were recorded. There were no differences in clinical symptoms of patients and controls. The research was carried out in conformity to the Declaration of Helsinki principles and was approved by the Ethics Committee of Tongji Medical College of Huazhong University of Science and Technology.

### 2.2. Angiographic Review

All angiograms of the patients were reviewed by 2 experienced investigators without knowledge of clinical or lab findings and a consensus was reached as to the diagnosis. Significant coronary artery stenosis was defined as stenosis of at least 50% in a major epicardial vessel at any site.

Coronary ectasia was defined as dilatation of a coronary artery to 1.5 times or more the normal adjacent coronary vessel segment according to Falsetti and Carroll [7]. The ratio of the diameter of ectasia segment to that of the adjacent normal coronary segment was defined as the ectasia ratio.

To evaluate the extent of coronary ectasia in any of the affected major epicardial arteries (left anterior descending—LAD, right coronary artery—RCA, and left circumflex artery—LCx), the length and maximum diameter of the ectasia segment and the diameter of adjacent normal vessel segment were measured using Siemens angiography software. Where there were more than one ectatic segment in the vessel, the total length of ectasia segments was got by adding up the individual lengths of the ectatic segments.

TIMI frame count was determined in the ectatic right coronary arteries of patients with ectasia and coexisting CAD and also in the RCA of the control group patients, according to methods first described by Gibson et al. [8].

## 3. Classification of Coronary Ectasia

Ectasia patients were classified according to the Markis classification system which categorizes coronary ectasia into 4 types [9]: type I: diffuse ectasia in two or three vessels; type II: diffuse ectasia in one and localized ectasia in another vessel; type III: diffuse ectasia in one vessel; type IV: localized ectasia.

### 3.1. Statistical Analysis

All analyses were performed using SPSS statistical analysis software version 20. All continuous variables were expressed as mean ± SD. Pearson's correlation or spearman's test was performed to investigate the correlation of different parameters as appropriate. Independent* t*-test was used to compare means between groups, and Fisher's exact test was used to analyze differences in prevalence of clinical features between groups. A *P* value <0.05 was considered statistically significant.

## 4. Results

### 4.1. Prevalence of Coronary Ectasia among Patients Undergoing Angiography

Of the 789 patients examined, 30 were found to have coronary ectasia, a prevalence of 3.8%. Of these, 25 patients had coronary ectasia with associated obstructive coronary artery disease (83.3%), and 5 patients had isolated coronary ectasia (16.7%). There was a higher prevalence of coronary ectasia among males than females (25 males versus 5 females).

### 4.2. Patient Clinical Characteristics

The clinical characteristics of subjects with ectasia and CAD, isolated ectasia, and controls are summarized in Table 1.

There was no significant difference in prevalence of diabetes mellitus, hypertension, and smoking between isolated ectasia and ectasia with CAD patients, *P* > 0.05 (two-sided Fisher's exact test). Likewise, there was no difference in prevalence of diabetes and hypertension between controls and patients with ectasia and CAD and controls and patients with isolated ectasia, *P* > 0.05 (two-sided Fisher's exact test). However, the prevalence of smoking was significantly higher among isolated ectasia patients (80%) compared to controls (16.7%), *P* = 0.028 (2-sided Fisher's exact test), and among ectasia and CAD patients (56%) compared to controls (16.7%), *P* = 0.035 (2-sided Fisher's exact test).

### 4.3. Vessel Distribution of Coronary Ectasia

Most of the coronary ectasia occurred in the right coronary artery. Among the 25 patients with ectasia and CAD, ectasia vessel involvement was 70.4% in the RCA, 11.1% in the LAD, and 18.5% in the LCx (Table 1). In those with isolated ectasia, the RCA, LAD, and LCx were equally involved (33.33%), with one case having both LAD and RCA involvement (Table 1).

**Figure 1:**
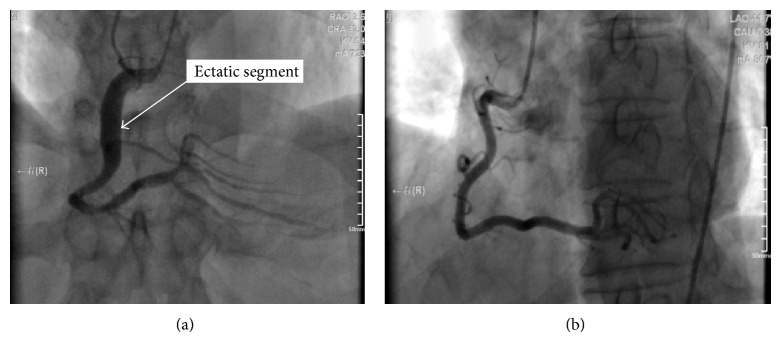
(a) Coronary angiogram showing right coronary ectasia. (b) Normal right coronary angiogram.

Figures 1(a) and 1(b) show an ectatic right coronary artery and a normal right coronary artery, respectively.

### 4.4. Effect of Ectasia on Coronary Flow

25 patients had coronary ectasia and coexisting obstructive coronary artery disease present. Of these, 19 (70.4%) had right coronary artery ectasia (Table 1). Because of the small number of patients with CAE occurring in the LAD (3 times) and LCx (5 times), we chose to use the right coronary artery for evaluation of the TIMI frame count and its correlation with ectasia size and length. Similarly, patients with isolated ectasia were few, with only 2 cases for each vessel affected, and thus we did not use this group for evaluation of effects of ectasia size and length on TIMI frame count. Among the patients whose RCA were examined for TFC, there was no noted intraluminal lesion in the ectasia segments.

Compared to the controls, the TFC in the RCA of ectasia patients with CAD was significantly increased (mean TFC = 16.83 ± 5.5 versus 23.84 ± 10), *P* = 0.035. There was a positive correlation, of moderate strength, between the ectasia size and TIMI frame count (TFC), with *r*(17) = 0.598, *P* = 0.007 (Table 2). Ectasia length however had a weak correlation with the TFC with a Spearman's rho (17) = 0.334, which was not statistically significant, *P* = 0.163 (Table 2).

We also measured the TFC in the right coronary arteries of isolated ectasia patients (2 cases), mean = 49.32 ± 10.9 (Table 1), although the number of cases was small to be used to make any statistical comparisons and conclusions.

### 4.5. Coronary Ectasia Type

Using the Markis classification of coronary ectasia, we found 1 patient (4%) had type I ectasia, 8 patients (32%) had type III ectasia, and 16 patients (64%) had type IV ectasia among ectasia patients with coexisting stenosis. Among the patients with isolated ectasia, type III was the most prevalent with 3 patients (60%) having this type, while 2 patients (20%) had type IV (Table 1).

## 5. Discussion

Coronary ectasia is a low prevalence but not absent disease among patients undergoing coronary angiography. Though a neglected and often incidental finding, research about this disease is still warranted and beneficial for proper diagnosis, assessment, and management of these patients.

As assessed from the right coronary artery, the TIMI frame count was significantly higher among patients with ectasia and CAD compared to controls (*P* = 0.03). This significant slow flow in ectatic coronary arteries has been reported by other investigators [5, 6]. The slow flow may be in part attributed to vascular dysfunction in the ectatic coronary arteries [10, 11]. Blood flow in the coronary arteries is not a passive process but has been shown to be an active process dependent on proper coronary vascular function [12]. Abnormal endothelial function is one of the factors leading to deranged vascular function inevitably leading to slowing of coronary flow [11, 13].

In addition to abnormal endothelial function, destruction of the extracellular matrix in coronary ectasia by matrix metalloproteinases (MMP) is another implicated cause of vascular dysfunction [14–17]. Dogan et al. found increased MMP3 and MMP9 in association with coronary ectasia [15]. Dahi et al. demonstrated induction of ectasia by increased levels of MMP2 [16]. Taken together, these factors lead to slow coronary flow, a factor which may predispose ectasia patients to ischemia and infarction. Indeed patients with increased ectasia size have been shown to be more prone to cardiac ischemia, and the degree of ischemia correlates with the ectasia size [18]. Moreover we have demonstrated that ectasia size is positively correlated with the TIMI frame count *r*(17) = 0.598, *P* = 0.007, with increased ectasia size being associated with slower coronary blood flow.

Added to this risk of slow flow is the fact that there is reported increase in platelet activity in coronary ectasia. Yasar et al. in their study done among patients with isolated ectasia reported increase in platelet activity among these patients [19]. These factors combined, compound the risk of coronary stasis and thrombosis in ectasia patients. It is reasonable to suggest that being more prone to thrombosis, ischemia, and infarction, patients with larger ectasia sizes should receive more aggressive treatment including surgery if necessary.

We noted in our study that there was no statistically significant correlation between the total length of ectasia and the TFC (*P* = 0.163). Although this was the case, it is worth noting that the extent of vessel involvement has been found to influence level of ischemia, with diffuse vessel ectasia predisposing patients more to ischemia compared to localized vessel involvement [20].

Based on the Markis classification, type IV was found to be the most common type of ectasia among patients undergoing angiography, with 16 patients (64%) having this form, 8 patients (32%) having type III, and only one patient (4%) having type 1, among those with ectasia and coexisting coronary artery disease. Of the 5 patients with isolated ectasia, Markis type III occurred in 60% of the cases while type IV occurred in 40% of cases. Classification of patients according to this system may have prognostic implications. Indeed Sayin et al. demonstrated that patients with diffuse vessel involvement are more prone to developing ischemia than those with localized vessel involvement [20]. Thus patients with diffuse vessel involvement may be at higher risk.

Among our study limitations is the relatively small number of patients and controls used for analysis. We however believe that our findings are representative and useful in trying to further understand and characterize this relatively rare disease entity.

## 6. Conclusion

In conclusion, we found that CAE has a prevalence of 3.8% among patients undergoing coronary angiography, and Markis type IV is the most common. Larger ectasia sizes are associated with slower coronary flow and thus patients with larger ectasia sizes may require more aggressive treatment approaches, probably including PCI and surgery.

## Figures and Tables

**Table 1:** Clinical characteristics of patients with coronary ectasia and control subjects with normal coronary arteries.

	Ectasia with CAD (*n* = 25)	Isolated coronary ectasia (*n* = 5)	Controls (*n* = 12)
Mean age (years)	67.2 ± 12.2	62.2 ± 11.51	59.7 ± 8.97

Sex (male/female)	24/4	4/1	5/7

Diabetes	7 (28%)	1 (20%)	2 (16.7%)

Smoking	14 (56%)	4 (80%)	2 (16.7%)

Hypertension	15 (60%)	2 (40%)	4 (33.3%)

Mean TFC in right coronary artery	23.84 ± 10	49.32 ± 10.9	16.83 ± 5.5

Mean ectasia length (mm)	34.68 ± 27.46	36.64 ± 18.17	N/A

Mean ectasia size (mm)	6.1 ± 1.66	8.1 ± 1.78	N/A

Ectasia ratio	1.72 ± 0.21	1.79 ± 0.27	N/A

Markis type	Type 1 = 1 Type 2 = 0 Type 3 = 8 Type 4 = 16	Type 1 = 0 Type 2 = 0 Type 3 = 3 Type 4 = 2	N/A

Vessel affected by ectasia	RCA = 19 LCX = 4 LAD = 2	RCA = 2 LCX = 2 LAD = 2	N/A

N/A = not applicable.

**Table 2:** Correlation of ectasia size and length with TIMI frame count (TFC) in the right coronary artery.

	TFC
Ecatsia_size	
Pearson correlation	0.598^**^
*P* value (2-tailed)	0.007
Number of cases	19
Ectasia length	
Spearman's rho correlation coefficient	0.33
*P* value (2-tailed)	0.16
Number of cases	19

^**^Correlation is significant at the 0.01 level (2-tailed).
